# Functional Segregation of Human Brain Networks Across the Lifespan: An Exploratory Analysis of Static and Dynamic Resting-State Functional Connectivity

**DOI:** 10.3389/fnins.2020.561594

**Published:** 2020-12-08

**Authors:** Benjamin M. Rosenberg, Eva Mennigen, Martin M. Monti, Roselinde H. Kaiser

**Affiliations:** ^1^Department of Psychology, University of California, Los Angeles, Los Angeles, CA, United States; ^2^Department of Psychiatry and Psychotherapy, University Hospital Carl Gustav Carus, Technische Universität Dresden, Dresden, Germany; ^3^Department of Psychology and Neuroscience, University of Colorado Boulder, Boulder, CO, United States

**Keywords:** development, resting state networks, functional connectivity, brain dynamics, brain imaging

## Abstract

Prior research has shown that during development, there is increased segregation between, and increased integration within, prototypical resting-state functional brain networks. Functional networks are typically defined by static functional connectivity over extended periods of rest. However, little is known about how time-varying properties of functional networks change with age. Likewise, a comparison of standard approaches to functional connectivity may provide a nuanced view of how network integration and segregation are reflected across the lifespan. Therefore, this exploratory study evaluated common approaches to static and dynamic functional network connectivity in a publicly available dataset of subjects ranging from 8 to 75 years of age. Analyses evaluated relationships between age and static resting-state functional connectivity, variability (standard deviation) of connectivity, and mean dwell time of functional network states defined by recurring patterns of whole-brain connectivity. Results showed that older age was associated with decreased static connectivity between nodes of different canonical networks, particularly between the visual system and nodes in other networks. Age was not significantly related to variability of connectivity. Mean dwell time of a network state reflecting high connectivity between visual regions decreased with age, but older age was also associated with increased mean dwell time of a network state reflecting high connectivity within and between canonical sensorimotor and visual networks. Results support a model of increased network segregation over the lifespan and also highlight potential pathways of top-down regulation among networks.

## Introduction

The organization of the human brain includes distinct functional brain networks that are implicated in different cognitive functions (Fox et al., 2005). Prior work has demonstrated that resting-state functional connectivity (rsFC) can be used to identify several canonical brain networks that are reliably observed within and between individual subjects (Damoiseaux et al., 2006). Studies of functional neurodevelopment have since examined how the brain’s functional connections change over the course of aging, as these changes may be especially relevant to psychopathology, neurodegeneration, or normative cognitive decline.

Two dimensions of rsFC may contain complementary information about intrinsic properties of network functioning: static rsFC and dynamic rsFC. The most basic distinction between these terms is that static rsFC refers to properties that do not vary over time, whereas dynamic rsFC refers to properties that vary over time or capture variance in static rsFC over time (although other definitions of dynamic rsFC reserve this term only for properties that capture the temporal sequence of data-points; see Liégeois et al., 2017). Although there is considerable debate regarding how best to disentangle meaningful dynamic signal from measurement noise (Hindriks et al., 2016), dynamic properties may represent the processes by which network form, dissolve, and interact with one another over time (Hutchison et al., 2013; Kaiser et al., 2016).

Within the static rsFC literature, prior research has suggested a pattern of neurodevelopment characterized by network “segregation,” wherein short-range connections between separable canonical resting-state networks (RSNs) become less coordinated, as well as network “integration,” wherein long-range connections within canonical RSNs tend to become more coordinated, particularly along the anterior-posterior axis (Dosenbach et al., 2010; Grayson and Fair, 2017). While adolescence has been identified as a crucial period concerning the development of functional integration and segregation (Stevens, 2016), functional segregation appears to decrease later in adulthood (Betzel et al., 2014; Chan et al., 2014).

In addition to the general patterns of RSN development described above, changes in particular RSNs have been highlighted in prior research on neurodevelopment, particularly among nodes of the prototypical default-mode (DN), frontoparietal (FN), salience (SN), and sensory networks. Broadly, static connectivity between nodes of different RSNs appears to decrease with aging, with some of the most pronounced changes involve sensorimotor regions and the precuneus (a structure spanning the FN and DN) (Allen et al., 2011). However, many changes in static connectivity have been highlighted within specific developmental periods. For instance, while segregation of sensorimotor regions appears to occur in childhood, segregation of task-positive networks such as the FN and SN continues throughout the transition from childhood into adulthood (Gu et al., 2015). Convergent findings further indicate that static connectivity specifically within the DN decreases over the course of advanced aging (Andrews-Hanna et al., 2007; Damoiseaux et al., 2007; Koch et al., 2010), whereas connectivity among regions of the DN, FN, and SN appears to strengthen from childhood into adulthood (Uddin et al., 2011; Gu et al., 2015), and then decrease in strength from early to late adulthood (He et al., 2013).

The extant literature relating development to dynamic rsFC within and between prototypical RSNs is far more limited, but early studies have provided insight into emerging directions in the field. For instance, while functional networks are generally preserved across the lifespan, young adults compared with children tend to show higher variability in rsFC, but lower variability in task-related functional connectivity during cognitive tasks (Hutchison and Morton, 2015). One interpretation is that these developmental changes in rsFC variability reflect either continued exploration of various brain states during early development (Deco et al., 2013), or the capacity for adult brains to suppress dynamic fluctuations when engaging in a cognitive task (Cohen, 2018). Interestingly, prior evidence also indicates that older adults tend to exhibit weaker connections throughout the brain, but this difference is primarily attributable to decreases in absolute rsFC rather than increases in rsFC variability (Tian et al., 2018). As these studies demonstrate, dynamic measures of functional connectivity can provide unique information about neurodevelopment over and above static connectivity measures.

Although research into neurodevelopment of functional networks has revealed promising insights, a number of important gaps remain. First, few prior studies have considered rsFC across the lifespan, instead tending to focus on specific periods of development. By leveraging data across a range of ages from childhood through late adulthood, it may be possible to detect age-related differences that occur on a longer timescale. Likewise, using such an approach may also highlight specific developmental periods most strongly associated with these changes. Second, the majority of rsFC studies focus on static or dynamic connectivity, rather than integrating multiple analytical methods into a common framework. As a result, the overlapping and distinct contributions of static and dynamic rsFC strategies remain largely understudied.

This exploratory project aimed to evaluate the relationships between age and static and dynamic rsFC in healthy individuals from the publicly available Human Connectome Project (HCP) Lifespan dataset (Van Essen et al., 2013). Strengths of this dataset include the broad age range, the high quality of data acquisition (functional neuroimaging data were collected at high temporal resolution, supporting dynamic analytic methods), and public availability of data (supporting open science goals and replication). Because data were drawn from an existing dataset, there are also some limitations (addressed further in the Discussion). Therefore, we emphasize the exploratory nature of these analyses. Analyses used complementary methods for interrogating rsFC: static rsFC, which we define as average connectivity across the scans, as well as dynamic rsFC, which we define as metrics of connectivity stability over time (e.g., variability in connectivity, persistence of network connectivity “states”). This study specifically aimed to compare the effects of age on functional network properties across these validated approaches. In light of the above discussion, we predicted that (1) older age would be related to higher static rsFC among regions within canonical RSNs and (2) older age would be related to lower static rsFC among regions in different canonical RSNs, particularly along the anterior-posterior axis. In addition, we hypothesized that (3) older age would be related to differences in rsFC variability and functional network dwell time. The latter dynamic hypotheses do not predict the direction of effects, because these methods are relatively new and there is mixed evidence regarding the direction of age-related effects on network dynamics. Because sex (Biswal et al., 2010; Allen et al., 2011; Zhang et al., 2018) and working memory task performance (Damoiseaux et al., 2007; Sala-Llonch et al., 2012) have been previously implicated in studies of rsFC, we included these variables as covariates in all analyses. Additional analyses evaluated the extent to which sex and working memory task performance moderate observed differences in static and dynamic rsFC across the lifespan.

## Materials and Methods

### Participants

Original data collection was approved by the Washington University Institutional Review Board. The HCP Lifespan Pilot (Van Essen et al., 2013) includes eight functional magnetic resonance imaging (fMRI) scans during eyes-open rest for 27 healthy individuals (15 female, 12 male) ranging from 8 to 75 years of age, organized into one of five age bins (8–9; 14–15; 25–35; 45–55; 65–75).

### Data Acquisition Parameters

Data were acquired on a 3-Tesla Siemens Connectome MRI scanner at Washington University in St. Louis, MO. Functional runs were acquired with a voxel resolution of 2 mm × 2 mm × 2 mm, 72 slices, using an 810 mm × 936 mm field of view. Each run was comprised of 420 frames using a repetition time (TR) of 0.72 s and an echo time (TE) of 33.2 ms. For each individual subject, the runs alternated phase encoding directions, such that the odd runs were in the left-to-right direction, whereas the even runs were in the right-to-left direction.

### Data Pre-processing

Unprocessed functional and structural MRI data corrected for gradient distortion were downloaded directly from the HCP website (Marcus et al., 2011). Our pre-processing pipeline was implemented in SPM12. Images were realigned using the default least squares approach in SPM12. Due to the rapid acquisition parameters (*TR* = 0.72 s, multi-band factor = 8) of these data, slice-timing correction was not applied (Glasser et al., 2013). Data were then spatially normalized into the standard Montreal Neurological Institute (MNI) space (Friston et al., 1994), resliced to 2 mm × 2 mm × 2 mm voxels, and smoothed using a Gaussian kernel with a full-width at half-maximum (FWHM) of 6mm. At the first-level of analysis, outlier volumes were censored and motion regressors were included as first-level covariates. To further reduce spurious correlations in rsFC due to subject head motion (Power et al., 2013), the Artifact Detection Tools (ART)^1^ software was used to identify and statistically censor volumes of significant movement or signal spikes from individual fMRI runs, also on the first level of analysis.

The total number of outlier motion volumes was also calculated in ART to identify if individual subjects lost an excessive amount of data due to censoring, thereby warranting exclusion from statistical analysis. Using a threshold of ≥15% volumes censored across the four fMRI runs, no subjects were identified as motion outliers. To account for the effects of frame-by-frame head displacement on rsFC (Power et al., 2012), estimates of total framewise displacement from ART were averaged across the four runs and included as a covariate in the group level of analysis. As gray matter volume is known to change across the lifespan (Ge et al., 2002), it is possible that changes in gray matter could influence the results of the present study. Unfortunately, it was not possible to disentangle effects of age from effects of gray matter volume, given high collinearity between these variables in prior studies (Walhovd et al., 2011) and in the present sample (*r* = 0.9497, *p* < 0.0001) (see Supplement 1). However, this is an intriguing question for future research.

### Independent Components Analysis (ICA)

In preparation for our analyses investigating persistence of whole-brain functional connectivity states, we first performed independent components analysis (ICA) implemented in the Group ICA of fMRI Toolbox v3.0b (GIFT^2^; Calhoun, 2004) to create spatial maps of nodes that would be used in subsequent analysis. Concatenating across the four runs of rsfMRI for each participant, we used ICA to detect voxels that are temporally correlated with one another (compared with chance likelihood). Correlated voxels were then blindly separated using Infomax (Calhoun et al., 2001), an algorithm that was repeated 10 times in ICASSO (Himberg and Hyvarinen, 2003) on 200 subject-specific principal components in order to compile the data into statistically independent components. We implemented group information guided independent component analysis (Calhoun et al., 2001) to perform back-reconstruction, yielding subject-specific spatial maps and time-courses. Based on prior work (Allen et al., 2011), we selected 100 components as the final model order for the present analysis, thresholding each component at *Z* >3.5 to enhance spatial specificity.

We were specifically interested in functional networks that included spatial components overlapping with the AN, DN, FN, Somatomotor (SM), SN, and Visual (VIS) networks. Based on visual inspection of the components in MNI space by two independent raters (Kelly et al., 2010), we identified 38 components for analysis. The other 62 components were omitted because they either belonged to other RSNs (e.g., auditory) or they were deemed to reflect motion- or noise-related artifacts. Of the 38 components included for analysis, we sorted them into one of the six canonical networks-of-interest. For a visual representation of how these components compare to a functional atlas of canonical resting brain networks (Yeo et al., 2011), see Supplement 2.

### Primary and Exploratory Analyses

The HCP protocol includes resting-state fMRI scans following both structural and task-fMRI paradigms. In order to avoid contamination effects of task-related activation on subsequent rsfMRI scans, we included only the resting scans collected prior to task scanning (i.e., we included four of the eight rsfMRI scans for each subject) in the primary neuroimaging analyses. Our group-level variables were mean-centered Age (binned by: 8–9; 14–15; 25–35; 45–55; 65–75), contrast-coded Sex (male; female), mean-centered WM performance (% accuracy during the neutral N-Back Task), and Framewise Displacement (average framewise displacement across the four runs).

Exploratory analyses were conducted to evaluate replication of findings in the second series of resting data collected from the same subjects, after completing task neuroimaging. Although we focus our main analyses on pre-task resting scans (due to the potential confounding effect of prior task engagement), replication could support reliability of methods and age differences. As one subject only completed six of the eight fMRI runs, this subject was omitted from replication analyses. Statistically significant results from the primary analyses were examined for replicability at uncorrected *p* < 0.05.

### Moderating Variables: Sex and Working Memory (WM) Task Performance

All subjects completed an N-Back task using neutral images (places, tools, faces, and body parts). Averaging across 2-back and 0-back trials, mean-centered performance (% accuracy) on the N-Back task was included as a behavioral regressor in group-level general linear models investigating static and dynamic rsFC metrics. Of the 27 subjects included in the analyses, all but one subject performed at greater than 50% accuracy in the WM task, so that subject was omitted from analyses. Therefore, the final dataset included 26 subjects (14 female, 12 male).

Sex, working memory task performance, and framewise displacement were included as covariates in all models. Unless noted (see Supplement 6), there were no significant effects of these covariates on rsFC measures.

### Analytical Approaches

#### Static rsFC Analyses

Static, time-invariant analyses were implemented in the CONN functional connectivity toolbox (Whitfield-Gabrieli and Nieto-Castanon, 2012)^3^. Physiological noise was controlled with CompCor, an algorithm in which the timeseries of activation is extracted from subject-specific tissue masks (white matter, cerebrospinal fluid), and principal components analysis is applied to estimate physiological noise reflected in these timeseries, after which the resulting components are included as covariates in a denoising regression (for additional details on this approach, see Behzadi et al., 2007; Chai et al., 2012; Whitfield-Gabrieli and Nieto-Castanon, 2012). Finally, we applied a band-pass filter of 0.008–0.09 Hz to further remove high-frequency activity associated with physiological functioning (oscillations at a frequency higher than expected for functional brain data, e.g., respiratory and cardiac noise, see Cordes et al., 2001) and low-frequency activity associated with scanner drift.

One of the 38 components, centered on bilateral anterior insula, underwent additional editing in the MarsBaR Toolbox (Brett et al., 2002) to enhance anatomical specificity by removing midline structures. To test for lateralized effects among regions of interest (ROIs), 14 of the 38 components were split in MarsBaR, yielding separate left and right ROIs for these components. Therefore, a total of 52 ROIs were used in this analysis.

We then performed a general linear model to investigate main effects of Age, and effects of Age moderated by Sex, or WM performance, on static rsFC among the ROIs derived from ICA. Across all static rsFC models, significance testing in CONN was thresholded at a false-discovery rate (FDR) *p* < 0.05 to correct for multiple comparisons at the analysis-level (Benjamini and Hochberg, 1995).

#### Dynamic rsFC Analyses: Variability in rsFC (vFC)

For analysis of dynamic variability in functional connectivity between individual ROIs, we utilized a sliding-window approach to measure the variability in ROI-ROI connectivity (vFC) (for examples, see Kaiser et al., 2016; Pelletier-Baldelli et al., 2018). To complete vFC, we entered the same 52 ROIs in the CONN toolbox. Initial denoising (for motion and physiological noise) was identical to that above, for static rsFC, but the band-pass filter was set at 0.0224–0.09 Hz to remove high-frequency activity characteristic of physiological noise and remove low-frequency activity with a period that is greater than the duration of each sliding window (Leonardi and Van De Ville, 2015). Sliding windows were calculated using a window length of 44.64 s and sliding the onset of each window by 3.6 s for a total of 72 windows (see Supplement 3 for details on how these parameters were selected; of note, the same sliding window size was used for GIFT analyses, below). Within each sliding window, we computed a Pearson’s correlation between individual seeds and all other seeds, yielding a single 52 × 52 correlation matrix of Fisher-transformed correlation coefficients (beta values) for each window and for each participant. To capture dynamic variability in rsFC over time, we computed the standard deviation of the beta values (SD_*b*_) across matrices for each ROI-to-ROI correlation coefficient, for each participant. This calculation yielded a matrix of ROI-to-ROI SD_*b*_ values for each participant. We then performed a general linear model to investigate main effects of Age, and effects of Age moderated by Sex or WM performance, on these SD_*b*_ values for each ROI with every other ROI. Across all dynamic rsFC models, significance testing was thresholded at a false-discovery rate (FDR) *p* < 0.05 to correct for multiple comparisons at the analysis-level (Benjamini and Hochberg, 1995). These corrections were performed in MATLAB 2015b using the fdr_bh function (written by David Groppe).

#### Dynamic rsFC Analyses: Intrinsic Functional Connectivity States

For analysis of dynamic network functioning as operationalized by functional connectivity states, we implemented the time-varying approach developed by Allen et al. (2014) and Rashid et al. (2014, 2016). First, to remove low-frequency noise, such as scanner drift, motion artifacts, and other sources of variance that may not be captured during ICA, component time-courses underwent post-processing using a fifth-order Butterworth low-pass filter with a high frequency cutoff of 0.15Hz. Outliers were removed based on the median absolute deviation using 3dDespike in AFNI (Cox, 1996) and were replaced using a third-order spline fit to the clean time-courses.

After ICA post-processing, the subject-specific spatial maps were then analyzed using a sliding-window procedure identical to the steps described in vFC, with a window length of 44.64 s and sliding the onset of each window by 3.6 s. Next, within each sliding window, pair-wise Pearson’s correlations were performed between each spatial component and all other spatial components from the ICA, yielding a 38 × 38 correlation matrix for each sliding window.

These correlation matrices (across windows and participants) were partitioned via *k*-means clustering, a data-driven method to cluster the average correlations over time into a discrete number of categories. Our approach adhered to the standard settings for *k*-means clustering within the GIFT Toolbox (Allen et al., 2014; additional information about the implementation of *k*-means clustering can be found in the GIFT Toolbox manual^4^). At a given time-point, each correlation matrix was categorized into one discrete “intrinsic connectivity network state” (ICN state), which represents a pattern of brain functional connectivity across ROIs. The present analysis yielded four ICN states (with the number of clusters (*k*) determined using the elbow criterion of the cluster validity index). The categorization of a correlation matrix into one discrete ICN state was assigned based on the likelihood that it resembled one ICN state compared to other ICN states (for details, see Allen et al., 2014; Rashid et al., 2014). For ease of interpretability, the matrices displaying each network state have been organized so that spatial components are grouped in adjacent rows/columns according to prior canonical RSNs.

Next, brain activity was classified into one of the four states at a given time-point. Then, we calculated the average time spent in each state before switching to another state (mean dwell time; MDT). We then performed a general linear model to investigate main effects of Age, and effects of Age as moderated by Sex and WM performance, on MDT in a particular ICN state.

#### Supplementary Analyses

Based on current standards, the three analytical approaches described above employed slightly different pre-processing methods to investigate static and dynamic rsFC (specifically with regard to temporal filtering). Therefore, we repeated the static and MDT analyses to match the temporal filtering used in the vFC approach (see Supplements 7, 8 for additional information).

## Results

**FIGURE 1:**
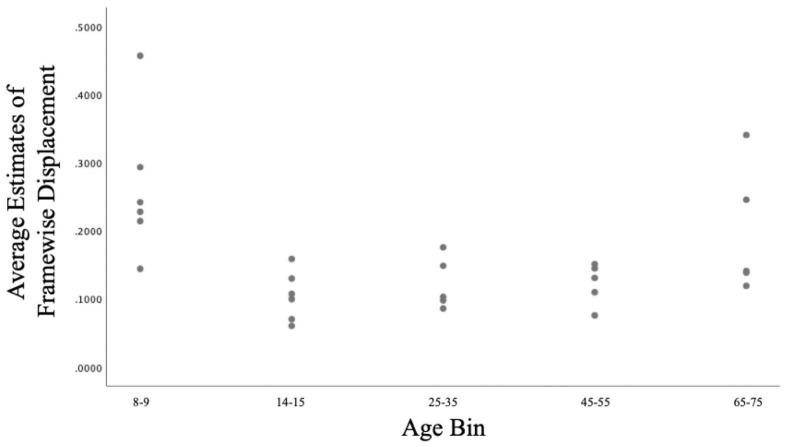
Scatterplot of the relationship between Age and Framewise Displacement in the current sample.

Age was not significantly correlated with Framewise Displacement (*R*^2^ = 0.044, *p* = 0.295). However, the youngest (ages 8–9) and oldest (ages 65–75) subjects tended to exhibit greater movement than did subject from other age bins (see Table 1 and Figure 1).

**TABLE 1:** Sex, age, and average framewise displacement values for each subject in the HCP Lifespan dataset.

HCP ID	Sex	Age bin	Estimate of average framewise displacement (mm)
LS2001	F	8–9	0.227275
LS2003	M	8–9	0.292575
LS2008	M	8–9	0.45585
LS2009	F	8–9	0.214069
LS2037	M	8–9	0.241275
LS2043	F	8–9	0.143725
LS3017	F	14–15	0.060225
LS3019	M	14–15	0.1583
LS3026	F	14–15	0.129525
LS3029	F	14–15	0.06995
LS3040	F	14–15	0.10705
LS3046	F	14–15	0.0994
LS4025	M	25–35	0.148225
LS4036	M	25–35	0.097575
LS4041	F	25–35	0.08575
LS4043	F	25–35	0.1028
LS4047	F	25–35	0.1752
LS5007	F	45–55	0.1444
LS5038	M	45–55	0.1506
LS5040	M	45–55	0.109475
LS5041	F	45–55	0.13035
LS5049	M	45–55	0.075475
LS6003	M	65–75	0.33965
LS6006	M	65–75	0.118725
LS6009	F	65–75	0.13815
LS6046	M	65–75	0.140525

### Static rsFC Analyses

General linear models first investigated main effects of Age (controlling for Sex, WM Performance, and Framewise Displacement) and the moderating effects of Sex and WM Performance in predicting static rsFC among pairs of the 52 ICA-derived components.

**FIGURE 2:**
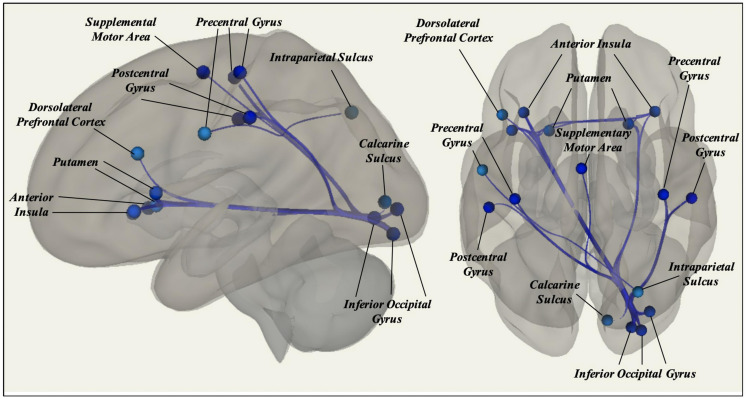
Significant effects of Age on static rsFC controlling for Gender, WM Performance, and Framewise Displacement, with resting-state functional connectivity tending to decrease among ROIs in different canonical functional networks.

**FIGURE 3:**
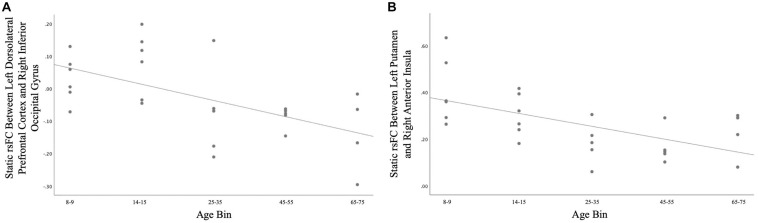
**(A)** Age differences in static rsFC between right inferior occipital gyrus and left dorsolateral prefrontal cortex [*t*(21) = −4.01, *p* = 0.00063]. Of note, similar patterns were observed for the effect of age on static rsFC between other ROIs in visual systems and ROIs in other canonical networks. **(B)** Age differences in static rsFC between left putamen and right anterior insula [*t*(21) = −3.92, *p* = 0.00075]. Similar age effects were detected between other ROIs in the canonical affective network and ROIs in the canonical salience network.

A total of 26 ROI-to-ROI pairs exhibited a significant main effect of Age. All of these effects were negative, including decreasing connectivity of AN ROIs with the SN as well as decreasing connectivity of VIS ROIs with ROIs in the AN, FN, SM, and SN (see Figure 2 and Table 2 for list of effects, see Supplementary Table S4a for average connectivity values within each age bin). Further inspecting these effects, positive rsFC between these ROIs tended to exhibit a positive-to-negative change in connectivity over the course of aging, particularly involving the right inferior occipital gyrus ROIs (see Figure 3A). However, four ROI-ROI pairs became less positive across the lifespan (see Figure 3B): the left putamen (of the AN) with the right anterior insula (of the SN), the right putamen (of the AN) with the right anterior insula (of the SN), and the right putamen (of the AN) with the left anterior insula (of the SN, two separate ROIs). There were no main or interactive effects regarding Sex and WM Performance.

**TABLE 2:** Significant effects of Age in predicting static rsFC between ROIs, grouped by network.

Effects of age on static rsFC of AN ROIs with SN ROIs		Resting-state series 1 (Pre-task scans)		Resting-state series 2 (Post-task scans)	
AN component	SN component	T-statistic	*p*-value	T-statistic	*p*-value
Left putamen	Right anterior insula	*t*(21) = −3.94	0.00075	*t*(20) = −3.92	0.0009
Right putamen	Left anterior insula (a)	*t*(21) = −5.29	0.00003	*t*(20) = −2.86	0.0097
Right putamen	Left anterior insula (b)	*t*(21) = −5.46	0.00001	*t*(20) = −3.26	0.0039
Right putamen	Right anterior insula	*t*(21) = −5.12	0.00005	*t*(20) = −3.06	0.0062

**Effects of age on static rsFC of AN ROIs with VIS ROIs**		**Pre-task scans**		**Post-task scans**	
**AN component**	**VIS component**	**T-statistic**	***p*-value**	**T-statistic**	***p*-value**

Left putamen	Right calcarine sulcus	*t*(21) = −4.00	0.00065	*t*(20) = −2.69	0.0140
Left putamen	Right inferior occipital gyrus (a)	*t*(21) = −4.53	0.00018	*t*(20) = −3.75	0.0013
Left putamen	Right inferior occipital gyrus (c)	*t*(21) = −4.93	0.00007	*t*(20) = −5.27	0.00002
Right putamen	Right inferior occipital gyrus (a)	*t*(21) = −5.04	0.00003	*t*(20) = −2.80	0.0111
Right putamen	Right inferior occipital gyrus (c)	*t*(21) = −4.37	0.00027	*t*(20) = −3.42	0.0027

**Effects of age on static rsFC of FN ROIs with VIS ROIs**		**Pre-task scans**		**Post-task scans**	
**FN component**	**VIS component**	**T-statistic**	***p*-value**	**T-statistic**	***p*-value**

Left dorsolateral prefrontal cortex	Right inferior occipital gyrus (c)	*t*(21) = −4.01	0.00063	*t*(20) = −0.98	0.3372

**Effects of age on static rsFC of SM ROIs with VIS ROIs**		**Pre-task scans**		**Post-task scans**	
**SM component**	**VIS component**	**T-statistic**	***p*-value**	**T-statistic**	***p*-value**

Left postcentral gyrus	Right inferior occipital gyrus (c)	*t*(21) = −4.48	0.00021	*t*(20) = −1.52	0.1436
Left precentral gyrus (a)	Right inferior occipital gyrus (b)	*t*(21) = −4.78	0.00010	*t*(20) = −3.34	0.0033
Left precentral gyrus (a)	Right inferior occipital gyrus (c)	*t*(21) = −5.82	0.00001	*t*(20) = −2.78	0.0177
Left precentral gyrus (b)	Right intraparietal sulcus	*t*(21) = −4.75	0.00011	*t*(20) = 0.89	0.3857
Right postcentral gyrus	Right inferior occipital Gyrus (a)	*t*(21) = −3.97	0.00070	*t*(20) = −1.68	0.1094
Right postcentral gyrus	Right inferior occipital gyrus (c)	*t*(21) = −4.62	0.00015	*t*(20) = −2.02	0.0567
Right precentral gyrus	Right inferior occipital gyrus (a)	*t*(21) = −4.03	0.00060	*t*(20) = −2.27	0.0347
Right precentral gyrus	Right inferior occipital gyrus (b)	*t*(21) = −5.29	0.00003	*t*(20) = −2.48	0.0223
Right precentral gyrus	Right inferior occipital gyrus (c)	*t*(21) = −4.25	0.00036	*t*(20) = −1.98	0.0620
Supplementary motor area	Right inferior occipital gyrus (c)	*t*(21) = −4.27	0.00034	*t*(20) = −1.81	0.0858

**Effects of age on static rsFC of SN ROIs with VIS ROIs**		**Pre-task scans**		**Post-task scans**	
**SN component**	**VIS component**	**T-statistic**	***p*-value**	**T-statistic**	***p*-value**

Left anterior insula (a)	Right inferior occipital gyrus (a)	*t*(21) = −4.16	0.00044	*t*(20) = −1.86	0.0780
Left anterior insula (a)	Right inferior occipital gyrus (c)	*t*(21) = −5.16	0.00004	*t*(20) = −2.98	0.0074
Left anterior insula (b)	Right inferior occipital gyrus (a)	*t*(21) = −4.81	0.00009	*t*(20) = −2.84	0.0102
Left anterior insula (b)	Right inferior occipital gyrus (b)	*t*(21) = −4.89	0.00008	*t*(20) = −2.00	0.0587
Left anterior insula (b)	Right inferior occipital gyrus (c)	*t*(21) = −6.06	0.00001	*t*(20) = −3.84	0.0010
Right anterior insula	Right inferior occipital gyrus (a)	*t*(21) = −3.87	0.00089	*t*(20) = −3.04	0.0064

*AN, Affective Network; DN, Default Network; FN, Frontoparietal Network; SM, Sensorimotor; SN, Salience Network, VIS, Visual. The letters (a), (b), and (c) denote regions for which multiple ROIs were used. Primary analyses focusing on the pre-task scans were corrected for multiple comparisons at an analysis-level FDR of p < 0.05. For significant connectivity pathways that survived FDR correction, exploratory analyses examined these pathways in the post-task scans at uncorrected p < 0.05.*

### Dynamic rsFC Analyses: Variability in rsFC (vFC)

General linear models investigated main effects of Age (controlling for Sex, WM Performance, and Framewise Displacement) and the moderating effects of Sex and WM Performance in predicting the SD_*b*_ for each ROI-to-ROI pair. Significance testing was thresholded at a false-discovery rate *p* < 0.05 for each set of linear models.

No ROI-to-ROI pairs exhibited a significant main effect of Age in predicting SD_*b*_ of rsFC across sliding-windows. Several ROI-to-ROI pairs exhibited non-significant main effects of Age on SD_*b*_ that failed to survive FDR correction (see Supplement 5 for details). There were no significant main or interactive effects of Sex or WM Performance, controlling for other covariates.

### Dynamic rsFC Analyses: Intrinsic Functional Connectivity States

General linear models first investigated the main effects of Age (controlling for Sex, WM Performance, and Framewise Displacement) in predicting MDT of the four GIFT-derived network states. Although Sex and WM performance were also included as moderators, no moderated effects emerged across any group-level analyses (see Supplement 6 for main effects of these variables). Significance testing was thresholded at a false-discovery rate *p* < 0.05 to correct for four comparisons using the same procedure described above (*p*-crit = *r* × 0.05/n).

#### State 1

**FIGURE 4:**
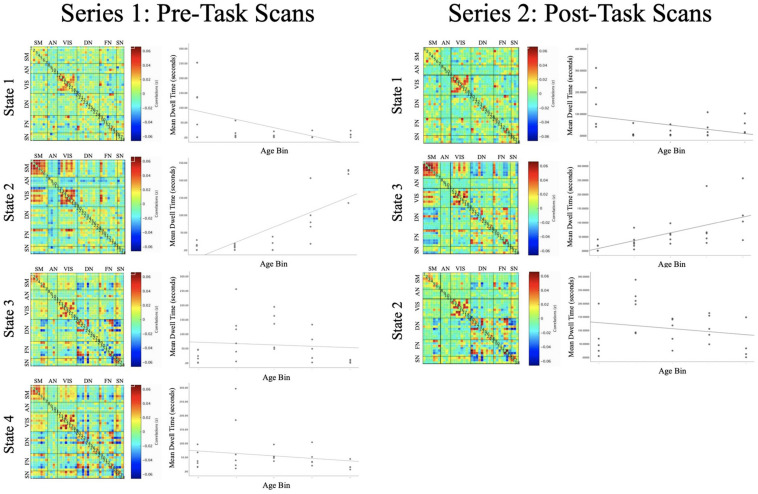
Each of the intrinsic functional connectivity states are organized into a correlation matrix, with ROIs grouped by functional domain for pre-task **(left)** and post-task **(right)** functional runs. Scatterplots depict the association between Age and Mean Dwell Time for each of the ICN states. State 2 **(left)** and State 3 **(right)** exhibited similar profiles of connectivity, with Mean Dwell Time tending to increase across the lifespan [*t*(20) = 3.780, *p* = 0.001; *t*(19) = 3.326, *p* = 0.003].

Intrinsic connectivity network (ICN) State 1 was characterized by pronounced coordination among regions of the VIS (see Figure 4). This ICN state also included positive rsFC between the dorsomedial prefrontal cortex (of the DN) and the rostral anterior cingulate cortex (of the FN), the bilateral hippocampus (of the AN) and the rostral anterior cingulate cortex (of the FN), and between the superior parietal lobule (of the FN) and the intraparietal sulcus (of the VIS), as well as negative rsFC (anticorrelations) between the ventral striatum (of the AN) and the dorsal anterior cingulate cortex (of the SN) and between the bilateral inferior frontal gyrus (of the FN) and the superior parietal lobule (of the FN).

FDR-corrected regression analyses revealed a significant main effect of Age predicting MDT of State 1 [*t*(20) = -3.494, *p* = 0.002], such that younger subjects tended to exhibit greater MDT in this ICN state (see Supplementary Table S4b for average MDT values within each age bin).

#### State 2

State 2 was defined by positive rsFC within and between regions of the Sensorimotor and VIS networks, except for negative rsFC (anticorrelations) between the bilateral inferior occipital gyrus (in the VIS) with SM regions (see Figure 4). This functional network state was also defined by positive rsFC between regions of the SM and VIS networks with the superior parietal lobule (of the FN) and by negative rsFC between the ventral striatum (of the AN) with the bilateral anterior insula (of the SN).

FDR-corrected regression analyses revealed a significant main effect of Age predicting MDT of State 2 [*t*(20) = 3.780, *p* = 0.001] such that older subjects tended to exhibit greater MDT of this ICN state (see Supplementary Table S4b for average MDT values within each age bin).

#### State 3

ICN State 3 was defined by positive rsFC within the SM and within the VIS, as well as a mixture of positive and negative rsFC among regions of the DN, FN, and SN (see Figure 4).

FDR-corrected regression analyses did not reveal a significant main effect of Age predicting MDT of State 3 [*t*(21) = -1.072, *p* = 0.296].

#### State 4

ICN State 4 was defined by positive rsFC within the SM and within the VIS, as well as a mixture of positive and negative rsFC among regions of the DN, FN, and SN (see Figure 4).

One subject was omitted from regression analysis because their MDT for State 4 was a significant outlier (>3 standard deviations above the mean). FDR-corrected regression analyses did not reveal a significant main effect of Age predicting MDT of State 4 [*t*(20) = −0.700, *p* = 0.492]. Inclusion or removal of the outlier subject did not alter the significance of these results [*t*(21) = −0.616, *p* = 0.545].

### Exploratory Analyses

As noted above, exploratory analyses were performed to test replication of age effects in the second series of (post-task) resting-state scans. Only effects that were significant in the main experimental analyses (i.e., the first series of pre-task resting-state scans) were eligible for testing in these exploratory analyses.

#### Static rsFC Analyses

General linear models investigated main effects of Age (controlling for Sex, WM Performance, and Framewise Displacement) in predicting static rsFC among the 26 ROI-ROI pairs implicated by the primary analysis, in the second series of resting-state scans.

A total of 18 ROI-ROI pairs exhibited a significant main effect of Age in the replication dataset (included in Table 2). All of these effects were negative, as in the primary analysis. An additional four ROI-ROI pairs exhibited a non-significant trending main effect of Age, and four ROI-ROI pairs did not exhibit a replication of effects presented in the primary analysis.

#### Dynamic rsFC Analyses: Intrinsic Functional Connectivity States

The replication analysis in the second resting-state series yielded three ICN states, which showed connectivity profiles that were highly similar to those in the primary analysis (see Figure 4). General linear models investigated the main effects of Age (controlling for Sex, WM Performance, and Framewise Displacement) in predicting MDT of the three GIFT-derived network states.

In this exploratory replication analysis a significant main effect of Age was detected for State 3 [*t*(19) = 3.326, *p* = 0.003], an intrinsic connectivity network that was similar to State 2 from the primary analysis. As in the primary analysis, older subjects tended to exhibit greater MDT in this functional network state.

## Discussion

The present study leveraged a publicly available dataset to explore how several different aspects of brain network functioning may differ across age groups, ranging from childhood to older adulthood. We first conducted a static rsFC analysis to evaluate overarching patterns of functional connectivity over time between ROIs. We then analyzed the standard deviation of these correlations as a method for evaluating the variability in functional connectivity patterns over time. Finally, we evaluated average dwell time in particular functional connectivity “states” of coordinated brain activity before neural systems switch to another state. These approaches each provided a different measure of how brain networks function, which we then linked to age-related differences across the lifespan.

Considering static rsFC, associations between anterior and posterior ROIs in different canonical networks tended to exhibit a positive-to-negative shift across the lifespan. These effects notably involved correlations between ROIs in the VIS (especially the right inferior occipital gyrus) and ROIs in the FN, SN, and SM. Associations between posterior and subcortical ROIs in different canonical networks exhibited a similar positive-to-negative shift, notably involving correlations between the right inferior occipital gyrus and right calcarine sulcus (of the VIS) with the left and right putamen (of the AN). In contrast to anterior-posterior and posterior-subcortical findings, associations between anterior and subcortical ROIs tended to involve positive correlations that become less positive across the lifespan, rather than shifting from positive to negative associations. These effects notably involved correlations between the left and right putamen (of the AN) with the left and right anterior insula (of the SN).

Several of the static rsFC findings replicated in exploratory analyses that aimed to reproduce main effects (from resting-state scan series 1, pre-task) in a second dataset (resting-state scan series 2, post-task). Of the 26 ROI-ROI pairs that were significantly associated with Age in the primary analysis, 18 of these effects replicated and an additional four of these effects exhibited a (non-significant) trend in the same direction. Effects that did not replicate included age effects on connectivity between the right inferior occipital gyrus (of the VIS) the right and left postcentral gyrus (of the SM), the right intraparietal sulcus (of the VIS) and the left precentral gyrus (of the SM), and the right inferior occipital gyrus (of the VIS) with the right inferior frontal gyrus (of the FN).

There were no significant associations between Age and vFC, although non-significant associations suggested potential decreases in variability between anterior and posterior ROIs in different canonical networks (see Supplement 5). This contrasts with prior findings of increased rsFC variability in young adults compared with children and youth (Hutchison and Morton, 2015). However, as the present study assessed vFC differences across the lifespan, this study is better positioned to evaluate overarching lifespan changes in vFC and less suited to detect subtle changes that emerge during the transitions from childhood to adolescence and early adulthood. Furthermore, the small sample size employed in the present study may further limit its ability to detect such changes. Research aimed at understanding the precise association between vFC and Age within specific developmental periods, such as during adolescence, can address these limitations.

Converging with static rsFC patterns, the findings related to persistence of functional connectivity brain states (evaluated here with MDT) revealed that older subjects tended to spend more time dwelling in a state characterized by integration within and between VIS and SM ROIs (State 2). Of note, exploratory analyses provided evidence of replication in the second dataset collected from the same subjects (following task administration). In contrast, younger subjects tended to spend more time in a state characterized by less substantial integration among specifically VIS ROIs (State 1). However, these age effects were not replicated and should be interpreted with caution.

Interestingly, the tendency for older adults to dwell in a state defined by positive rsFC between SM and VIS ROIs may appear inconsistent with the results of static rsFC analyses, which showed decreasing overall rsFC between VIS and SM ROIs. However, this discrepancy also highlights the differences in age effects on standard static rsFC (which is based on overarching patterns of coordination) compared with dynamic measures of dwell time in transient rsFC networks (which are based on time spent in a particular state of rsFC, although patterns of rsFC may be quite different at other times in the scan). For example, these findings suggest that at older ages, SM and VIS ROIs tend to become less coordinated on average across an extended period of scanning; however, older brains also tend to persist longer in a brain state defined by increased integration among these systems once that brain state has been entered. These effects highlight the value of investigating and comparing age differences in static vs. dynamic properties of rsFC. A next step for this work may build on this comparative approach by developing methods that integrate static and dynamic properties in the same measurement or analyses.

Overall, the present findings support the theory that network boundaries become sharper across development (Fair et al., 2007; Grayson and Fair, 2017), evidenced by decreasing static rsFC among regions of distinct prototypical networks (Tian et al., 2018). The positive-to-negative shift in static rsFC among many of these ROIs suggests that network segregation may result in a qualitative shift in the relationship between regions, such that correlated regions not only disassociate from one another but may become anticorrelated later in life. Based on the present results, this phenomenon may especially emerge between regions of the visual system and other canonical networks, whereas regions of the affective and salience systems may simply become less correlated across the lifespan. Furthermore, age-related differences in MDT tended to show increased time spent in states of integration within and between the SM and VIS networks. Many of these same pathways were implicated in the static rsFC analysis, suggesting that the combined use of static and dynamic approaches may reveal subtle features of functional brain connectivity across the lifespan.

### Limitations and Future Directions

We chose to test hypotheses in the HCP Lifespan dataset because of several notable strengths: e.g., the sample featured a broad age range, and data were collected at high temporal resolution (important for dynamic analytic methods). However, there are also some limitations which restrict the scope and generalizability of the present study.

First, although the present analyses were conducted to be as similar as possible in processing steps, it is important to note that we chose to accept some differences in processing in order to follow standard conventions for each method (see CONN^5^ and GIFT^4^ manuals). Specifically, this included some differences in low- and high-pass temporal filtering, differences in the number of ROIs included (i.e., splitting of bilateral components in the static and vFC analyses), and differences in artifact detection and removal (i.e., use of CompCor and ART in CONN, use of 3dDespike in GIFT). Future research should explore how differences in standard approaches to physiological denoising and temporal filtering (such as those implemented in CONN and GIFT, as well as other toolboxes) may affect estimates of static and dynamic functional connectivity.

Second, the sample size for this publicly available dataset is modest, limiting statistical power. As the present study constitutes an initial step in characterizing patterns of functional connectivity across the human lifespan, it will be necessary to replicate and extend these exploratory findings in larger studies with a lifespan age range. In larger samples, replication of linear effects may be tested, in addition to exploring non-linear effects of age that may highlight specific developmental periods as crucial windows of dynamic network development. Likewise, a critical next step will be to focus on patterns of functional connectivity within specific age ranges, utilizing large datasets from studies such as the HCP Development (ages 5–21), HCP Aging (ages 36+), and Adolescent Brain Cognitive Development (a longitudinal study from age 9–20) initiatives.

Third, in cases where individuals tend to dwell longer in rare ICN states, it would be especially interesting to understand the cognitive significance of such a pattern. As the present study included only one metric of cognitive functioning, future research should evaluate a wider range of cognitive tasks to better characterize the psychological correlates of ICN state persistence, and how these correlates differ across the lifespan.

Fourth, future research should further clarify the relationship between static rsFC and MDT approaches, especially considering findings that diverge across the modalities. Given the rapidly changing suite of methods for investigating resting-state functional connectivity (Calhoun et al., 2014), methods comparison is an important step for evaluating the unique vs. overlapping information that can be gained with these different techniques.

Finally, the risk of onset of specific psychiatric disorders varies over development (Paus et al., 2008), and may correspond with key changes in functional brain networks. Future clinical research may employ a similar combination of approaches to identify how disruptions in network integration and segregation relate to the emergence of psychopathology during development. In sum, we emphasize the exploratory nature of the present analyses, and the potential value of future replication and new directions for this work.

## Conclusion

In conclusion, the present study supports a framework of both network segregation and integration across the lifespan, wherein sensory networks tend to become more integrated with one another and more segregated from other canonical brain networks. Likewise, the present study supports the use of multiple analytical approaches to evaluate static and dynamic trends in functional connectivity during the resting-state.

## Data Availability Statement

All datasets generated for this study are included in the article/Supplementary Material. Further inquiries can be directed to the corresponding author.

## Ethics Statement

The studies involving human participants were reviewed and approved by Washington University Institutional Review Board. Written informed consent to participate in this study was provided by the participants or their legal guardian/next of kin.

## Author Contributions

BR: conceptualization, formal analysis, and writing. EM: methodology and software. MM: methodology and supervision. RK: conceptualization, methodology, supervision, and writing. All authors contributed to the article and approved the submitted version.

## Conflict of Interest

The authors declare that the research was conducted in the absence of any commercial or financial relationships that could be construed as a potential conflict of interest.

## Footnotes

1 http://www.nitrc.org/projects/artifact_detect

2 https://trendscenter.org/trends/software/gift/

3 http://www.nitrc.org/projects/conn

4 https://trendscenter.org/trends/software/gift/docs/v4.0b_gica_manual.pdf

5 https://web.conn-toolbox.org/resources/manual
