# Supplementary material for: Functional Segregation of Human Brain Networks Across the Lifespan: An Exploratory Analysis of Static and Dynamic Resting-State Functional Connectivity
Source: Front Neurosci. 2020 Dec 8;14:561594. doi: 10.3389/fnins.2020.561594 (PMC7752769; doi:10.3389/fnins.2020.561594)
Supplement: Supplementary file 1 [file Data_Sheet_1.PDF]

# *Supplementary Material*

## Supplement 1

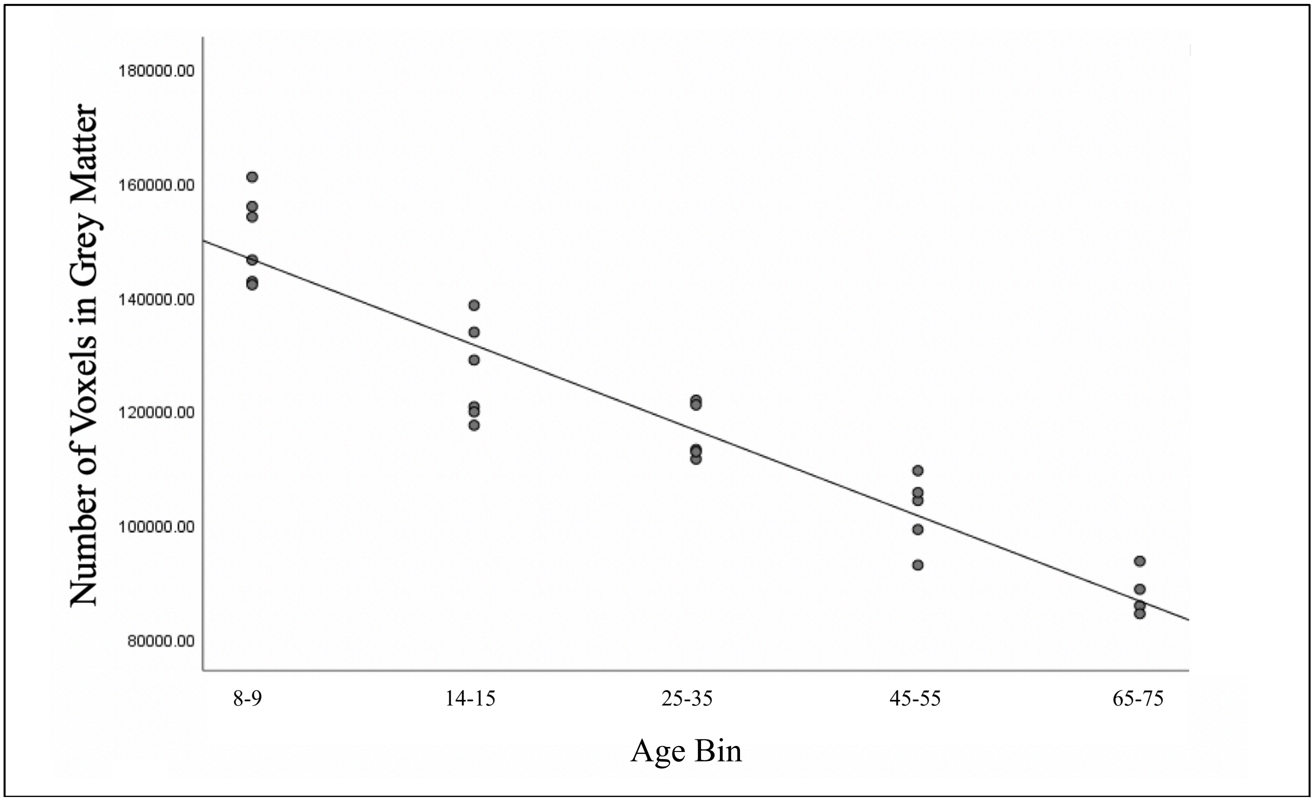


**Fig. S1:** The number of voxels in gray matter for each participant was highly correlated with Age (*r*=0.9497, *p*<0001).

**Supplement 2**

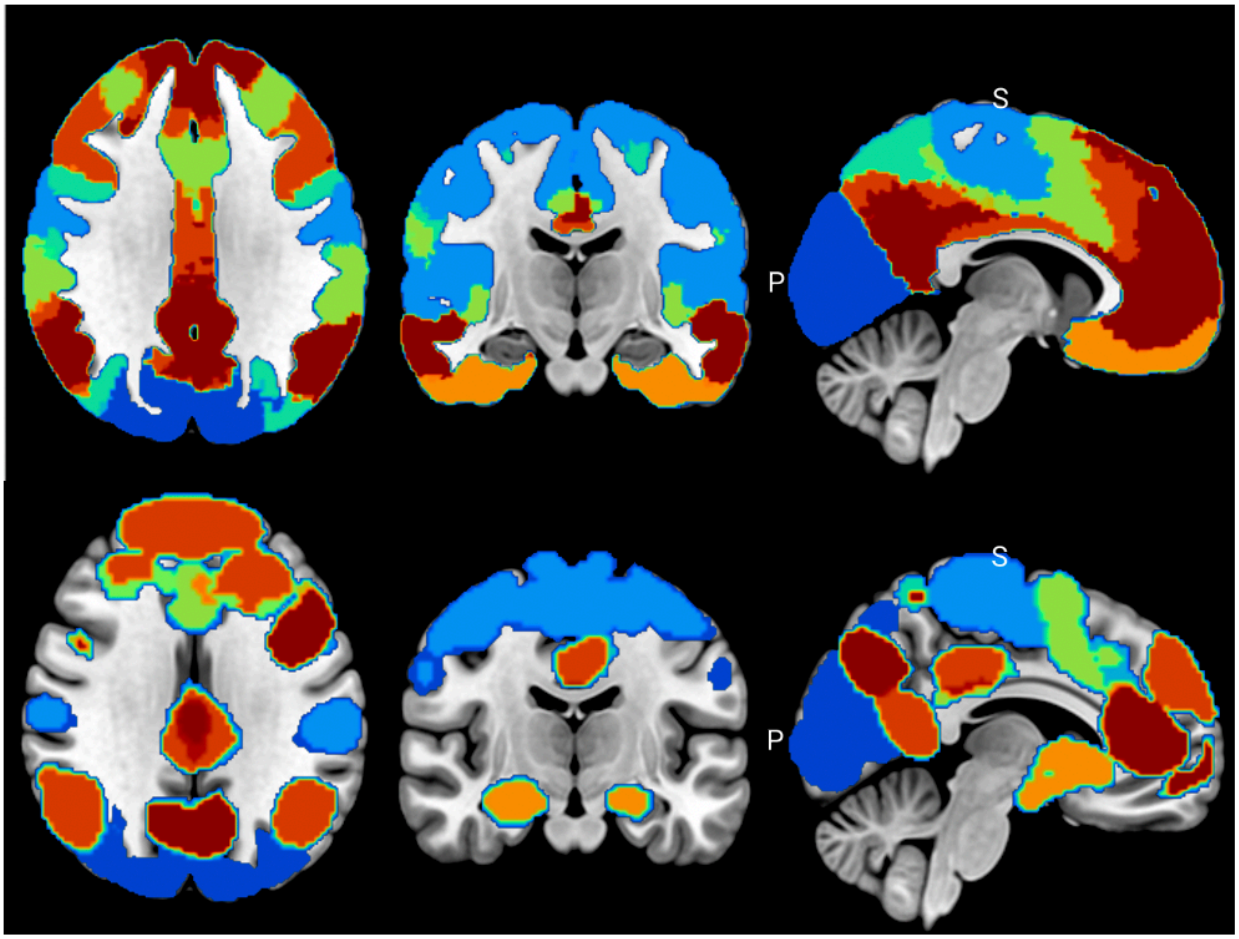


**Fig. S2:** Visual Representation of canonical resting-state brain networks derived from Yeo et al, 2011 (top) and regions of interest included in the present analyses that were derived from independent components analysis (bottom).

**Supplement 3**

Window length may impact the results of dynamic rsFC analyses, because window size determines the rsFC frequencies that contribute to within-window estimates of functional connectivity. Shorter windows may be more sensitive to rapid changes in connectivity, whereas longer windows may generate more reliable effects. To optimize this trade-off, window length is typically set between 30-60 seconds (Hutchison et al., 2013; Van De Ville et al., 2015, Kaiser et al., 2016). Step size may also impact estimates of dynamic functional connectivity, although to the authors' knowledge this has received less focus within the literature. The window length in this study was set to 5 TRs, or 3.6 seconds.

**Supplement 4**

| Effects of Age on Static rsFC of AN ROIs with SN ROIs | | Average Connectivity Value Per Bin (Fisher's Z) | | | | |
|---|---|---|---|---|---|---|
| **AN Component** | **SN Component** | *8 - 9* | *14 - 15* | *25 - 35* | *45 - 55* | *65 - 75* |
| Left Putamen | Right Anterior Insula | 0.4065 | 0.3028 | 0.1839 | 0.1656 | 0.2228 |
| Right Putamen | Left Anterior Insula (a) | 0.4886 | 0.3426 | 0.1721 | 0.1333 | 0.2583 |
| Right Putamen | Left Anterior Insula (b) | 0.4962 | 0.3707 | 0.1812 | 0.1634 | 0.2347 |
| Right Putamen | Right Anterior Insula | 0.6896 | 0.5812 | 0.3801 | 0.3889 | 0.4363 |
| | | | | | | |
| **Effects of Age on Static rsFC of AN ROIs with VIS ROIs** | | **Average Connectivity Value Per Bin (Fisher's Z)** | | | | |
| **AN Component** | **VIS Component** | *8 - 9* | *14 - 15* | *25 - 35* | *45 - 55* | *65 - 75* |
| Left Putamen | Right Calcarine Sulcus | 0.0985 | 0.1137 | 0.0474 | 0.0722 | 0.1578 |
| Left Putamen | Right Inferior Occipital Gyrus (a) | 0.1561 | 0.0849 | -0.0159 | 0.0045 | -0.1233 |
| Left Putamen | Right Inferior Occipital Gyrus (c) | 0.1694 | 0.1150 | -0.0052 | -0.0250 | -0.1177 |
| Right Putamen | Right Inferior Occipital Gyrus (a) | 0.1673 | 0.1188 | 0.0459 | 0.0185 | -0.0861 |
| Right Putamen | Right Inferior Occipital Gyrus (c) | 0.1796 | 0.1106 | 0.0018 | -0.0010 | -0.1345 |
| | | | | | | |
| **Effects of Age on Static rsFC of FN ROIs with VIS ROIs** | | **Average Connectivity Value Per Bin (Fisher's Z)** | | | | |
| **FN Component** | **VIS Component** | *8 - 9* | *14 - 15* | *25 - 35* | *45 - 55* | *65 - 75* |
| Left Dorsolateral Prefrontal Cortex | Right Inferior Occipital Gyrus (c) | 0.0311 | 0.0770 | -0.0740 | -0.0870 | -0.1358 |
| | | | | | | |
| **Effects of Age on Static rsFC of SM ROIs with VIS ROIs** | | **Average Connectivity Value Per Bin (Fisher's Z)** | | | | |
| **SM Component** | **VIS Component** | *8 - 9* | *14 - 15* | *25 - 35* | *45 - 55* | *65 - 75* |
| Left Postcentral Gyrus | Right Inferior Occipital Gyrus (c) | -0.0087 | 0.0583 | -0.0654 | -0.0846 | -0.3092 |
| Left Precentral Gyrus (a) | Right Inferior Occipital Gyrus (b) | 0.0347 | 0.1634 | 0.0984 | -0.0334 | -0.3303 |
| Left Precentral Gyrus (a) | Right Inferior Occipital Gyrus (c) | 0.0230 | 0.1716 | 0.1101 | -0.0357 | -0.3044 |
| Left Precentral Gyrus (b) | Right Intraparietal Sulcus | 0.1191 | 0.0578 | 0.0030 | 0.0001 | -0.0688 |
| Right Postcentral Gyrus | Right Inferior Occipital Gyrus (a) | 0.1557 | 0.1877 | 0.0904 | 0.0592 | -0.2086 |
| Right Postcentral Gyrus | Right Inferior Occipital Gyrus (c) | 0.0284 | 0.1344 | 0.0345 | 0.0138 | -0.3454 |
| Right Precentral Gyrus | Right Inferior Occipital Gyrus (a) | 0.1288 | 0.1809 | 0.1530 | 0.0416 | -0.1190 |
| Right Precentral Gyrus | Right Inferior Occipital Gyrus (b) | 0.0510 | 0.1188 | 0.1114 | -0.0082 | -0.2172 |
| Right Precentral Gyrus | Right Inferior Occipital Gyrus (c) | 0.0498 | 0.1386 | 0.1672 | -0.0127 | -0.2646 |
| Supplementary Motor Area | Right Inferior Occipital Gyrus (c) | 0.0480 | -0.0256 | -0.0122 | -0.0700 | -0.3377 |
| | | | | | | |
| **Effects of Age on Static rsFC of SN ROIs with VIS ROIs** | | **Average Connectivity Value Per Bin (Fisher's Z)** | | | | |
| **SN Component** | **VIS Component** | *8 - 9* | *14 - 15* | *25 - 35* | *45 - 55* | *65 - 75* |
| Left Anterior Insula (a) | Right Inferior Occipital Gyrus (a) | 0.1536 | 0.1556 | -0.0073 | 0.0004 | -0.1148 |
| Left Anterior Insula (a) | Right Inferior Occipital Gyrus (c) | 0.1118 | 0.1482 | -0.0199 | -0.0448 | -0.0811 |
| Left Anterior Insula (b) | Right Inferior Occipital Gyrus (a) | 0.1671 | 0.1411 | -0.0268 | 0.0102 | -0.1525 |
| Left Anterior Insula (b) | Right Inferior Occipital Gyrus (b) | 0.1759 | 0.0992 | -0.0082 | 0.0006 | -0.1221 |
| Left Anterior Insula (b) | Right Inferior Occipital Gyrus (c) | 0.1598 | 0.1346 | -0.0041 | -0.0016 | -0.0932 |
| Right Anterior Insula | Right Inferior Occipital Gyrus (a) | 0.2020 | 0.1208 | 0.0317 | 0.0065 | -0.1259 |

Table S4a: Average connectivity values within each age bin for ROI-ROI pairs that were significantly associated with Age (controlling for Sex, Working Memory Performance, and Framewise Displacement) in the static rsFC analysis. ROIs are grouped by network (AN = Affective Network; DN = Default Network; FN = Frontoparietal Network; SM = Sensorimotor; SN = Salience Network, VIS = Visual).

| **Effects of Age on MDT of ICN States** | **Average MDT Value Per Bin (seconds)** | | | | |
|---|---|---|---|---|---|
| | *8 - 9* | *14 - 15* | *25 - 35* | *45 - 55* | *65 - 75* |
| **State 1** | 135.9861 | 13.2708 | 5.5000 | 4.6000 | 9.9375 |
| **State 2** | 11.2813 | 8.2500 | 11.5667 | 93.7083 | 201.9510 |

Table S4b: Average Mean Dwell Time (MDT) values within each age bin for Intrinsic Connectivity Network (ICN) states that were significantly associated with Age (controlling for Sex, Working Memory Performance, and Framewise Displacement) during the ICN state analysis.

**Supplement 5**

Variability in functional connectivity (vFC) tended to decrease between ROIs (for examples, see Figs. 4a-b). In particular, the left dorsolateral prefrontal cortex exhibited decreasing vFC with the left and right calcarine sulci, left anterior insula, and dorsal anterior cingulated cortex / pre-supplementary motor area (see Table 2 for full list of effects).

| **Non-Significant Effects of Age on Variability in RSFC of FN ROIs with SN ROIs** | | | |
|---|---|---|---|
| **FN Component** | **SN Component** | **vFC T-Statistic** | **vFC *p*** |
| Left Dorsolateral Prefrontal Cortex | Dorsal Anterior Cingulate Cortex / Pre-Supplementary Motor Area | t(21) = -3.30 | *0.00339* |
| Left Dorsolateral Prefrontal Cortex | Left Anterior Insula | t(21) = -3.33 | *0.00319* |

| **Non-Significant Effects of Age on Variability in RSFC of FN ROIs with VIS ROIs** | | | |
|---|---|---|---|
| **FN Component** | **VIS Component** | **vFC T-Statistic** | **vFC *p*** |
| Left Dorsolateral Prefrontal Cortex | Left Calcarine Sulcus | t(21) = -3.50 | *0.00214* |
| Left Dorsolateral Prefrontal Cortex | Right Calcarine Sulcus | t(21) = -3.55 | *0.00191* |

| **Non-Significant Effects of Age on Variability in RSFC of SN ROIs with VIS ROIs** | | | |
|---|---|---|---|
| **SN Component** | **VIS Component** | **vFC T-Statistic** | **vFC *p*** |
| Right Anterior Insula | Left Intraparietal Sulcus | t(21) = -3.64 | *0.00153* |

**Table S5:** Non-significant FDR-corrected effects (uncorrected $p < .005$) of Age in predicting variability of rsFC between ROIs, grouped by network (FN = Frontoparietal Network; SN = Salience Network; VIS = Visual).

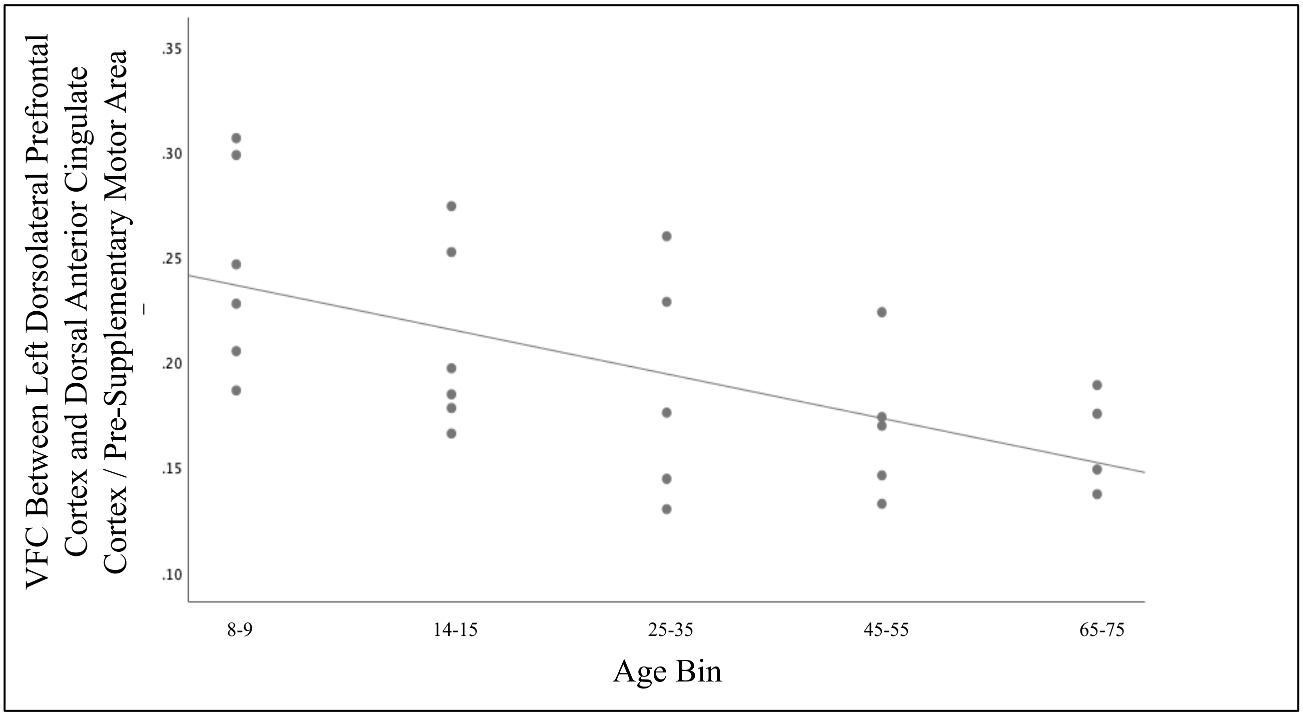


Fig. S5a: Decreasing vFC between the left dorsolateral prefrontal cortex and left anterior insula.

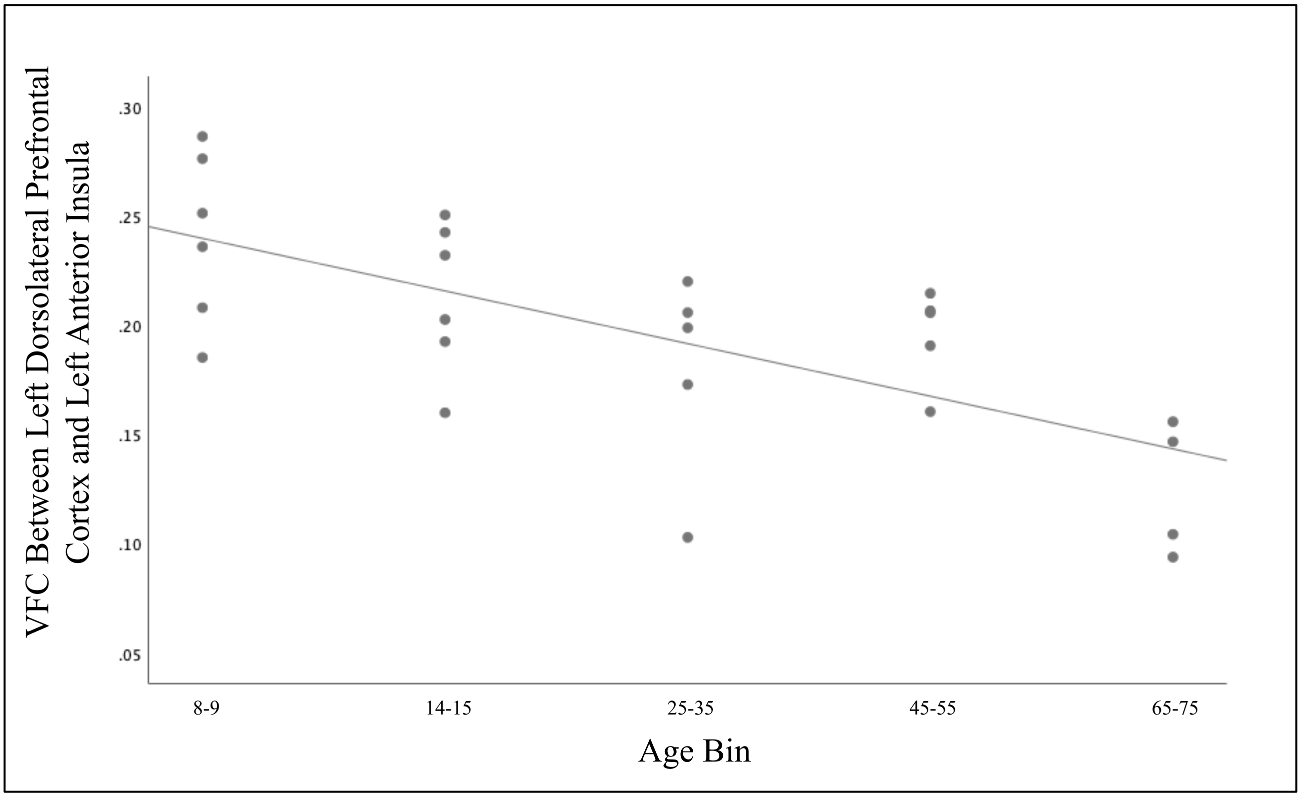


Fig. S5b: Decreasing vFC between the left dorsolateral prefrontal cortex and dorsal anterior cingulated cortex / pre-supplementary motor area.

**Supplement 6**

The main hypotheses controlled for Sex, Working Memory Performance, and Framewise Displacement as covariates, and these were not independent variables of interest. However, we report here if any of those variables had significant associations with MDT (controlling for other covariates in the models) in the ICN states from our main and exploratory analyses.

<u>Main Analysis with Resting-State Series 1 (Pre-Task Data)</u>

State 1:

- There was a significant main effect of Framewise Displacement ($t(20) = 2.438$, $p = .024$), such that subjects with greater head motion tended to exhibit greater MDT in this ICN state.

State 2:

- There was a significant main effect of WM Performance ($t(20) = -2.795$, $p = .011$), such that lower-performing subjects tended to exhibit greater MDT of this ICN state.

State 3:

- There was a significant main effect of Framewise Displacement ($t(20) = -2.203$, $p = .039$), such that subjects with less head motion tended to exhibit greater MDT in this ICN state.

State 4:

- There were no significant main effect of Sex, WM Performance, or Framewise Displacement in this ICN state.

<u>Exploratory Analysis with Resting-State Series 2 (Post-Task Data)</u>

State 1:

- There was a significant main effect of Sex ($t(19) = 2.445$, $p = .024$) and of Framewise Displacement ($t(19) = 6.055$, $p < .001$), such that male subjects and subjects with greater head motion tended to exhibit greater MDT in this ICN state.

State 2:

- There was a significant main effect of Framewise Displacement ($t(19)$ -3.320, $p = .003$), such subjects with less head motion tended to exhibit greater MDT of this ICN state.

State 3:

- There were no significant main effect of Sex, WM Performance, or Framewise Displacement in this ICN state.

**Supplement 7**

Modifying the band-pass filter had a modest effect on the results of the static rsFC analysis.

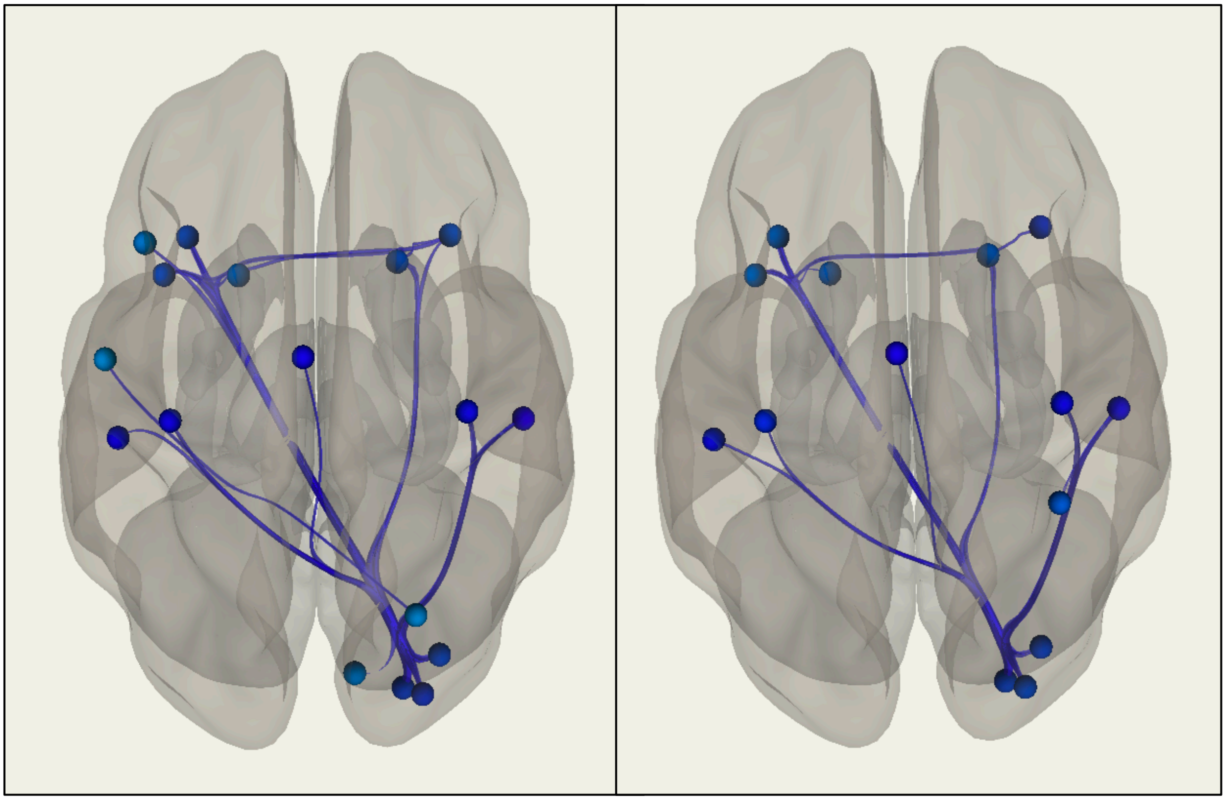

Fig. S7: Altering band-pass filtering from 0.008-0.09 Hz (left) to 0.0224-0.09 Hz (right) had a modest effect on the results of the static rsFC analysis. Age differences in static rsFC were largely consistent when considering a narrower frequency band, although some age differences dropped from significance.

**Supplement 8**

After modifying the band-pass filter, MDT analysis generated five network states (rather than the four listed in the main text). However, two of the new network states appeared comparable to States 1 and 2 of the original analysis and yielded similar effects of age.

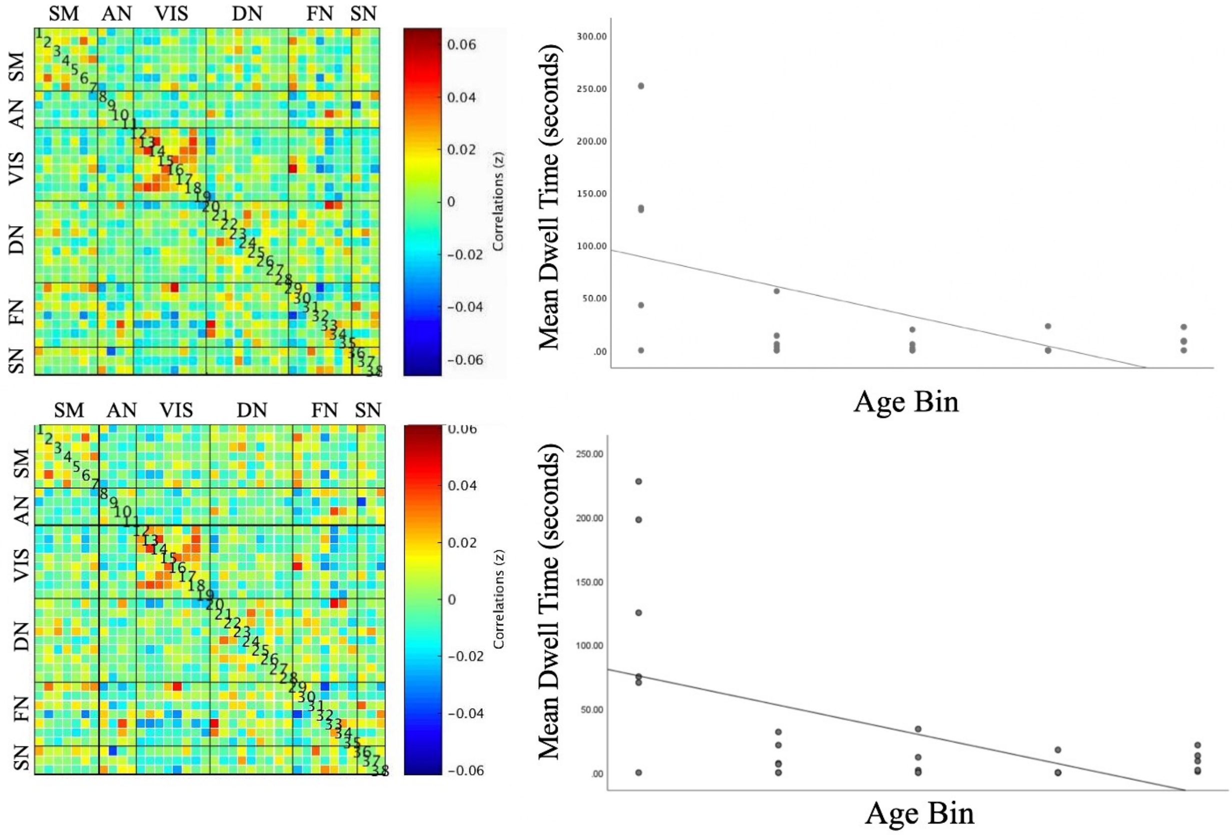


Fig. S8a: Altering high-frequency cutoff from 0.15 Hz (top) to 0.09 Hz (bottom) did not substantially impact the results of the State 1 MDT analysis.

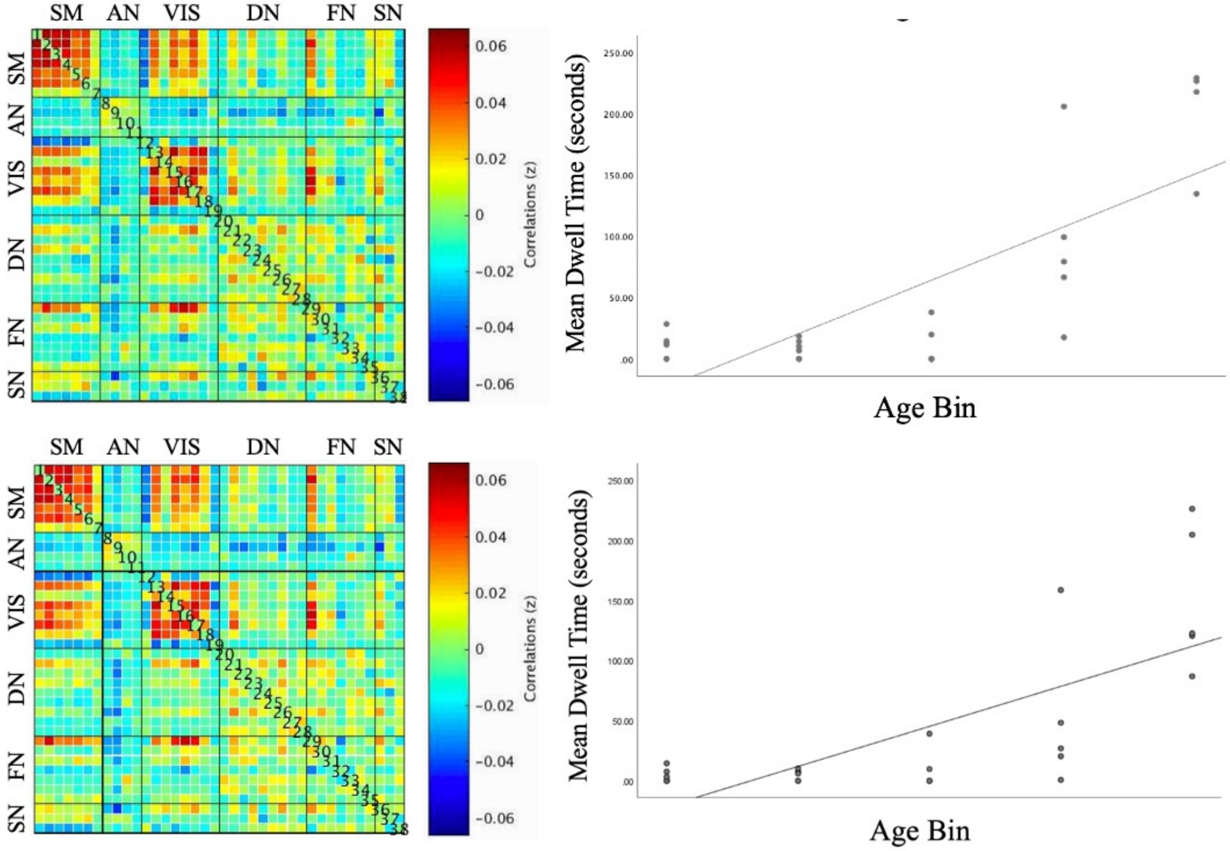


Fig. S8b: Altering high-frequency cutoff from 0.15 Hz (top) to 0.09 Hz (bottom) did not substantially impact the results of the State 2 MDT analysis.
